# Socio-economic inequalities in patient, primary care, referral, diagnostic, and treatment intervals on the lung cancer care pathway: protocol for a systematic review and meta-analysis

**DOI:** 10.1186/2046-4053-3-30

**Published:** 2014-03-25

**Authors:** Lynne F Forrest, Sarah Sowden, Greg Rubin, Martin White, Jean Adams

**Affiliations:** 1 Fuse, UKCRC Centre for Translational Research in Public Health, Newcastle University, Newcastle upon Tyne NE2 4AX, UK; 2 Institute of Health & Society, Newcastle University, Newcastle upon Tyne NE2 4AX, UK; 3 Wolfson Research Institute, Durham University, Queen’s Campus, Stockton on Tees TS17 6BH, UK

**Keywords:** Delay, Diagnosis, Interval, Lung cancer, Patient, Primary care, Referral, Socio-economic inequalities, Time, Treatment

## Abstract

**Background:**

Early diagnosis and treatment of cancer is thought to be important for improving survival. Longer time between the onset of cancer symptoms and receipt of treatment may help explain the poorer survival of UK cancer patients compared to that in other countries.

Socio-economic inequalities in receipt of, and time to, treatment may contribute to socio-economic differences in cancer survival. Socio-economic inequalities in receipt of lung cancer treatment have been shown in a recent systematic review. However, no systematic review of the evidence for socio-economic inequalities in time to presentation (patient interval), time to first investigation (primary care interval), time to secondary care investigation (referral interval), time to diagnosis (diagnostic interval), and time to treatment (treatment interval) has been conducted.

This review aims to assess the published and grey literature evidence for socio-economic inequalities in the length of time spent on the lung cancer diagnostic and treatment pathway, examining interim intervals on the pathway where inequalities might occur.

**Methods:**

Systematic methods will be used to identify relevant studies, assess study eligibility for inclusion, and evaluate study quality. The online databases of MEDLINE, EMBASE, and CINAHL will be searched to locate cohort studies of adults with a primary diagnosis of lung cancer; where the outcome is mean or median time to the interval endpoint (or a suitable proxy measure of this), or the likelihood of longer or shorter time to the endpoint; analysed by a measure of socio-economic position. Meta-analysis will be conducted if there are sufficient studies available with suitable data.

**Discussion:**

This review will systematically determine if there are socio-economic inequalities in time from symptom onset to treatment for lung cancer. If such inequalities are present, our review evidence will help inform the development of interventions to reduce the time to diagnosis and treatment, ultimately helping to reduce socio-economic inequalities in survival.

**Trial registration:**

PROSPERO CRD42014007145

## Background

**Figure 1:**
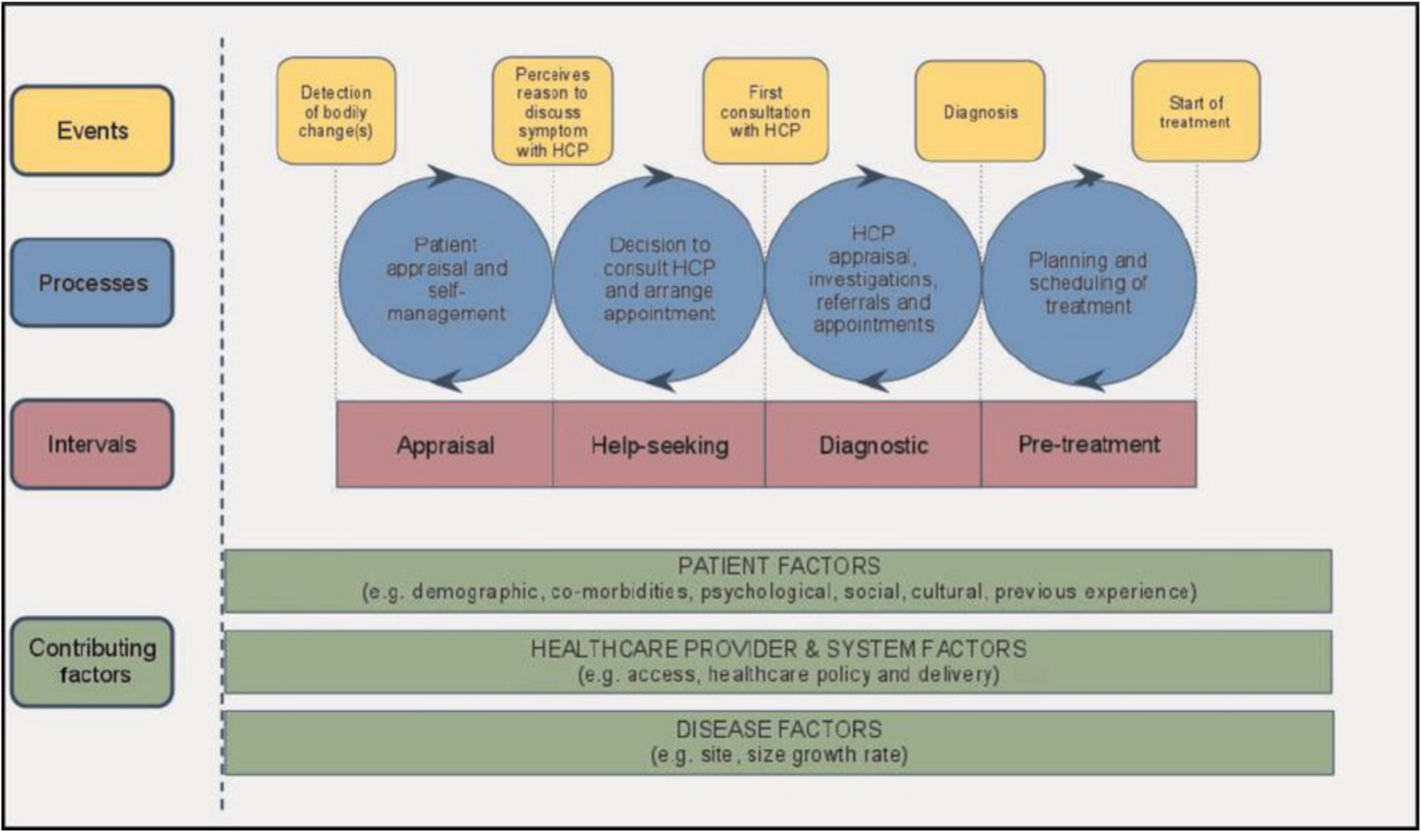
**Refined Anderson model of total patient delay **[6]**.**

**Figure 2:**
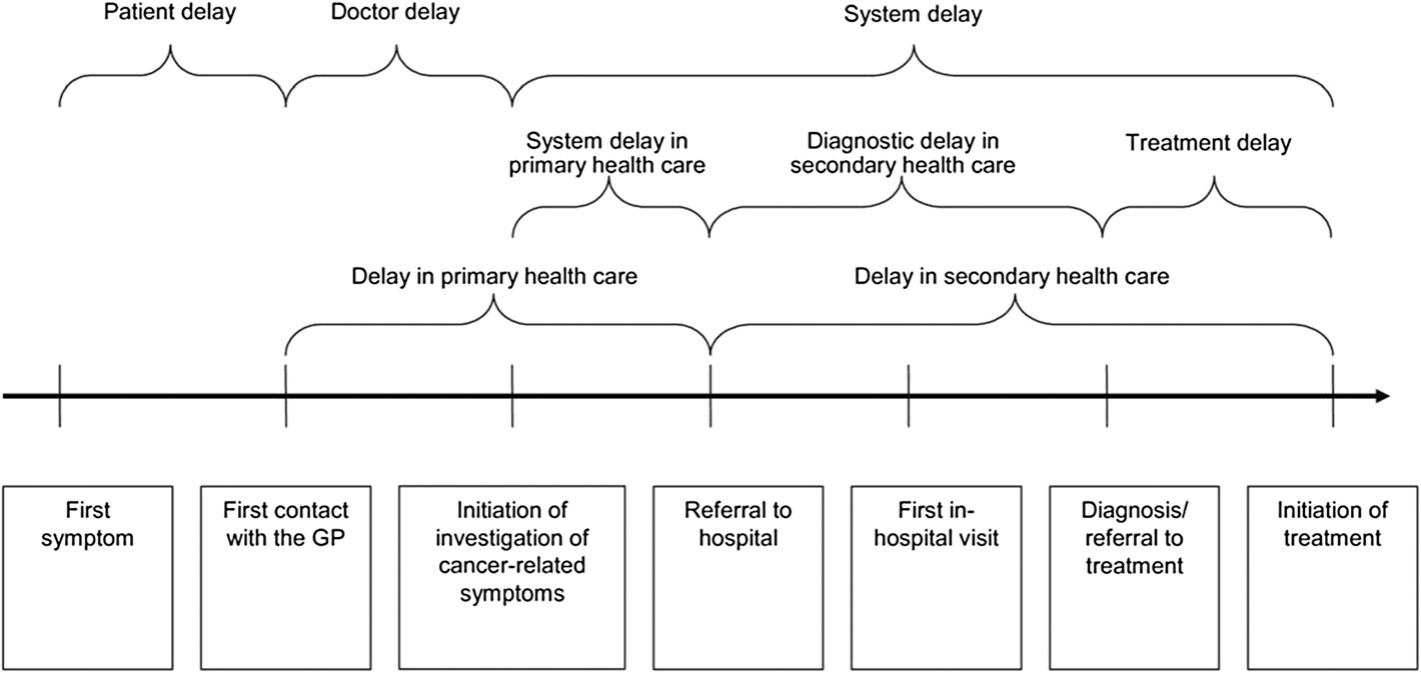
**Intervals on the cancer diagnosis and treatment pathway **[10]**.**

Lung cancer is the most common cancer worldwide. In the USA and the UK it is the second most incident cancer [1,2], as well as the most common cause of cancer mortality [2,3]. In the UK, fewer than 10% of those diagnosed with lung cancer survive for 5 years [4]. Longer time between the onset of cancer symptoms and receipt of treatment may contribute to the poorer survival of UK cancer patients compared to that found in other countries [5]. An early model of cancer delay, the Anderson model, attributed the majority of delays to patient factors but this has been updated to consider patient, tumour, and healthcare system factors (Figure 1) [6]. The following intervals on the care pathway have been identified: patient (incorporating appraisal and help-seeking intervals), primary care, referral, diagnostic, and treatment intervals [7-9], illustrated by a pathway model (Figure 2) [10]. Early diagnosis of cancer is thought to be important for improving outcomes, as survival is better for patients who are diagnosed at an early stage since they are more likely to be suitable for receipt of potentially curative treatment [5].

In 2000, the UK NHS Cancer Plan pledged to reduce delay in diagnosis and treatment and increase survival whilst acting to reduce inequalities [11]. In England, the National Awareness and Early Diagnosis Initiative is a scheme designed to encourage early presentation of patients to primary care and to improve general practitioner (GP) cancer recognition and referral. It proposes that delays may lead to diagnosis at a later disease stage and thus result in ‘potentially-avoidable’ deaths [5].

Intervention-generated inequalities are health inequalities that result from the way that health interventions are organised and delivered [12], so that although overall health may improve as the result of an intervention, differences in access to the intervention, differential uptake and delays in uptake might result in inequalities in outcome. Inequalities are likely to occur at many different stages of intervention pathways and act in a cumulative way [12].

A socio-economic gradient for lung cancer survival [13] is found in the UK. Socio-economic inequalities in receipt of lung cancer treatment have been shown in a recent systematic review and meta-analysis [14] and there is some evidence that inequalities in treatment contribute to socio-economic inequalities in survival [15]. It has also been suggested that inequalities in time to diagnosis and treatment might contribute to socio-economic differences in cancer survival [5]. However, there has been no systematic review of the evidence for socio-economic inequalities in time from onset of first symptom to treatment, and in the interim patient, primary care, referral, diagnostic, and treatment intervals on the lung cancer care pathway where inequalities might occur.

This review aims to investigate whether there are socio-economic inequalities in the length of time spent on the lung cancer diagnostic and treatment pathway and, if so, in which intervals (patient, primary care, referral, diagnostic, treatment) inequalities occur.

## Methods/design

Poorly defined definitions of the important time points that characterise pathway intervals have meant that it has previously been difficult to compare studies. An international consensus working group recently identified the following four time points as important: date of first symptom, date of first presentation, date of referral to secondary care, and date of diagnosis [7]. These can be used to construct defined time intervals with reference to both the Anderson (Figure 1) [6] and Hansen (Figure 2) [10] models of delay.

The following time intervals will be investigated: patient interval (time from the interval start point defined as date of first symptom, to the interval end point defined as date of first presentation); primary care interval (time from date of first presentation to date of first investigation); referral interval (time from date of GP referral to secondary care investigation); diagnostic interval (time from date of secondary care investigation to diagnosis OR time from GP referral to diagnosis); and treatment interval (time from diagnosis to treatment OR time from GP referral to treatment).

### Search strategy

Systematic methods will be used to identify relevant studies, assess study eligibility for inclusion, and evaluate study quality. A search will be undertaken to locate all studies published up to the date that the search is run, with a title and abstract published in English, examining differences, by socio-economic position (SEP), in patient, primary care, referral, diagnostic, and treatment time intervals on the care pathway for lung cancer. The searches will be re-run just before the final analyses and any further studies retrieved for inclusion.

One researcher (LF) will develop the search strategy, which will then be refined with the help of an Information Scientist and used to search the online databases of MEDLINE, EMBASE, and CINAHL. The search terms will be adapted for the different databases. Additional studies will be identified by reviewing the reference lists of relevant studies identified from the search and by using a forward citation search to identify more recent studies that have cited an older, relevant study. Grey literature including reports from cancer registries, lung cancer audit reports, published abstracts, and theses will also be searched for. EndNote software will be used to manage the references.

### Study eligibility

The following types of study will be eligible for inclusion:

• Cohort studies of adult participants who have a primary diagnosis of lung cancer (small-cell lung cancer or non-small-cell lung cancer – ICD10 C33 C34); published in a peer-reviewed journal or in the grey literature up to the date that the search is run; where the outcome is:

○ Mean or median time (days) to the interval endpoint (or a suitable proxy measure of this);

○ OR the likelihood (odds ratio or hazard ratio with 95% confidence intervals) of longer or shorter time to the endpoint;

○ AND where outcome is analysed by a measure of SEP (such as an individual or area-based measure of deprivation, poverty, income, or education).

The following are considered suitable proxy measures of length of time intervals on the pathway:

• Stage at diagnosis or stage at start of treatment [16]

• Type of referral (urgent vs. routine) [17]

• Emergency presentation [18]

• Number of pre-referral consultations [19]

The searches will be re-run just before the final analyses and any further studies retrieved for inclusion.

Preliminary independent screening of the titles and abstracts obtained from the database searches will be carried out by two researchers (LF and SS). Initial screening of titles will be carried out to remove obviously irrelevant papers. However, from a preliminary scoping review by LF, the early pilot searches recovered studies that, although they conducted analyses by SEP, did not always mention this in the abstract or title. Therefore, in the title search, any titles that refer to delay in, or time intervals for, lung cancer will be retained. Titles that consider disparities in cancer treatment will also be included as further checking of the abstract is required to see if inequalities in intervals on the care pathway to treatment are also examined.

Selected abstracts will then be screened and a subset of studies will be selected for further review and the full article obtained. Abstracts that refer to socio-economic inequalities in time intervals on the care pathway will be retained. Abstracts that refer to racial, ethnic, geographical, sex, and age-related time disparities as well as disparities by insurance type will also be retained as often these papers also look at SEP, even if this is not mentioned in the abstract. Two researchers (LF and SS) will then independently assess the selected full papers for eligibility according to the study-eligibility criteria detailed above. Any disagreements at any of the screening stages will be resolved by discussion between the two reviewers in the first instance. If agreement cannot be reached, then a third reviewer (JA) will independently review the title, abstract or full paper, as appropriate, and a majority decision will be taken on inclusion/exclusion.

### Data extraction

Data extraction will be carried out by LF and checked by SS using a pro-forma to be developed by LF for this purpose, based on previous review work [14]. Data relating to study authors, journal, study design, year of study, data source, population included, number of participants, years of diagnosis, time interval examined, measure of SEP, confounding variables included in the analysis (such as age, sex, stage, histology, histological subtype, co-morbidity, performance status, marital status, smoking status, cancer network, health board, hospital, distance from hospital or travel time, ethnicity, insurance status), outcome measures (mean or median time interval [or the proxy measure of time used]; odds ratio, hazard ratio), statistical tests carried out, significance (*P* values), precision (confidence intervals), and other variables that were significant, will be recorded.

There is evidence to suggest that insurance status is an important factor relating to access to lung cancer care in the US healthcare system [20] and so may impact on time-intervals on the care pathway. Therefore, as in a previous systematic review of intervention-generated inequalities [14], studies will be split into three categories: those carried out in a healthcare system free at the point of access (similar to the UK); those based on an insurance system (similar to the USA); those that include a mixture of free care and social insurance-based payment (some European systems).

### Study quality

Study quality will be appraised using a quality checklist based on that developed for a previous systematic review [14] and on the ‘Aarhus checklist’ [7], which has been developed to help assess the quality of studies that measure intervals on the cancer diagnostic and treatment pathway.

The following criteria will be used to assess study quality: study design, size, setting, dates, data sources, eligibility criteria, type of population included (including number of participants potentially eligible, number actually included, number analysed), missing or incomplete data reported, variables included (in terms of outcome, exposure, predictors, confounders), validity of the definition of time points and intervals, validity of the measure used to estimate time interval, validity of the measure of SEP, type of statistical analysis carried out, unadjusted and adjusted estimates reported, precision (confidence intervals), significance (*P* values) given, limitations of the study, potential bias addressed, and external validity of results.

### Statistical analysis

Random effects meta-analysis will be considered if there are sufficient studies which have the same type of outcome measure using the same comparator, across the same time interval. The I^2^ statistic will be used to assess heterogeneity. If it is not possible to conduct a meta-analysis, due to the heterogeneity of the studies, then narrative analysis will be carried out and the use of Harvest Plot methodology will be considered [21]. This is a method that has been devised for synthesising evidence from studies looking at the differential effects of interventions, where meta-analysis is not suitable.

Planned sub-group analyses by interval (patient, primary care, referral, diagnostic, and treatment intervals) and, within these intervals, by healthcare system category will be conducted. Studies reporting a proxy time interval measure will be analysed separately. Sensitivity analyses will be undertaken where required, for example, to examine the effect of including all potentially-eligible studies or only high quality studies in meta-analyses, or looking at both published and grey literature results compared to only those from published papers.

## Discussion

This review will systematically determine if there are socio-economic inequalities in time from symptom onset to treatment for lung cancer. If such inequalities are present, our review evidence will help inform the development of interventions to reduce the time to diagnosis and treatment, ultimately helping to reduce socio-economic inequalities in survival.

## Abbreviations

GP: General practitioner; SEP: Socio-economic position.

## Competing interests

We declare that we have no competing interests.

## Authors’ contributions

LF: Conception and design, data collection and analysis, manuscript writing and final approval of the manuscript. JA: Conception and design, critical revision and final approval of the manuscript. MW: Design, critical revision, and final approval of the manuscript. GR: Design, critical revision and final approval of the manuscript. SS: Design, data collection and analysis, critical revision and final approval of the manuscript. All authors read and approved the final manuscript.
